# Full-range (**VNIR–SWIR–MWIR–LWIR**) mineral and VNIR water spectra with co-located geochemistry from an acid mine drainage (AMD) site (Kirki, NE Greece)

**DOI:** 10.1038/s41597-026-07307-y

**Published:** 2026-05-12

**Authors:** Veronika Kopačková-Strnadová, Martin Kýhos, Jan Jelének, Marianthi Anastasatou, Pauliina Liwata-Kenttälä, Kati Laakso, Alexandros Liakopoulos, Constantinos Mavrogonatos

**Affiliations:** 1 Czech Geological Survey, Klárov 131/3, Malá Strana, 118 00 Praha 1, Czech Republic; 2 Hellenic Survey of Geology and Mineral Exploration, 1 Spirou Loui Str., Olympic Village, Acharnes P.C, 13677 Attiki, Greece; 3 Geological Survey of Finland, P.O. Box 77, FI-96100 Rovaniemi, Finland; 4 Geological Survey of Finland, P.O. Box 96, FI-02151 Espoo, Finland; 5 Department of Earth Sciences, University of Pisa, Via Santa Maria 53, 56126 Pisa, Italy

## Abstract

Acid mine drainage (AMD) environments host complex mineral–water systems that are not well represented by spectral libraries derived solely from pure minerals. We present an open dataset that integrates spectroscopy of natural AMD materials with co-located mineralogical and geochemical measurements from the Kirki (Saint Philippos) mine in NE Greece. The dataset includes: (i) laboratory mineral reflectance spectra spanning 350–15,375 nm (visible–near infrared (VNIR)–shortwave infrared (SWIR)–mid-wave infrared (MWIR)–longwave infrared (LWIR)) for 26 compositionally diverse solid samples; (ii) *in situ* water VNIR spectra from 337–823 nm collected over AMD-impacted streams and lakes; (iii) co-located field measurements (e.g., temperature, pH, electrical conductivity, turbidity); and (iv) an extensive suite of laboratory analyses for both solids and waters, including mineralogical, major-element, and trace-element data. All spectral and analytical products are provided in a harmonized format, with detailed metadata, instrument specifications, and processing documentation. The dataset is designed to support the development and testing of spectral analysis methods, quantitative retrieval algorithms, and cross-sensor comparisons in AMD settings, and to serve as a high-fidelity analog resource for planetary spectroscopy where sulfate- and Fe-oxide detection and longwave infrared mineral characterization are of interest.

## Background & Summary

Pure mineral spectral libraries have underpinned remote sensing, exploration, and planetary science by providing controlled reference spectra to identify diagnostic features, calibrate algorithms, and select endmembers for unmixing across visible–near infrared (VNIR)–short-wave infrared (SWIR)–long wave infrared (LWIR) spectral ranges. They enable attribution of absorption bands to specific vibrational and electronic transitions and support rule-based and derivative methods used in tools like Tetracorder^1–3^. However, real-world targets—such as soils, mine wastes, and altered rocks—rarely behave like ideal single-mineral samples. Intimate mixing, grain-size distributions, coatings, hydration, and oxidation state introduce nonlinear effects that can shift, broaden, or suppress diagnostic features, thereby reducing the fidelity of matches to pure endmembers.

Natural-sample (mixture-based) spectral libraries have been developed to address this gap by capturing more realistic spectral behavior and radiative-transfer effects observed in the field^4–7^. Such libraries can reproduce bandwidth and continuum characteristics for key diagnostic features (e.g., sulfate ν₃ near 8.5–9.5 µm; Al–OH and carbonate features in the SWIR and LWIR) and incorporate particle-size and texture dependencies that strongly influence reflectance and emissivity^7^. These properties are particularly relevant for applications that require robust classification, quantitative retrievals, and transferability across sensors and viewing geometries^8–10^. In practice, many workflows therefore employ a hybrid strategy: pure-mineral spectra are used to anchor diagnostic features, and rigorously characterized natural-sample spectra, together with independent mineralogical and chemical data (e.g., X-ray diffraction (XRD) / X-ray fluorescence (XRF), are used to refine and validate interpretations.

Acid mine drainage (AMD) environments are a prominent example where such complex spectral behaviour is expected. When iron sulfide–bearing rocks—especially pyrite (FeS₂)—are exposed to air and water, oxidation produces sulfuric acid and mobilizes metals; subsequent neutralization and weathering precipitate secondary iron-bearing minerals such as jarosite, schwertmannite, and goethite^11^. These processes can impart characteristic yellow to red staining and degrade water quality. Imaging spectroscopy can detect diagnostic features of both sulfates and these secondary alteration phases across VNIR–SWIR and into the TIR, but mapping in AMD-impacted terrains benefits from spectral libraries that reflect the mixed, hydrated, and weathered conditions typical of such sites.

Although AMD-related mineral spectroscopy in the VNIR–SWIR (400–2,500 nm) has been widely studied, and existing spectral libraries have been successfully applied to AMD mapping^12–14^, there is still no open-access, natural-sample spectral library for AMD mineral assemblages that is paired with comprehensive geochemical characterization. Knowledge gaps are even greater in the LWIR, where the spectral behaviour of AMD-related mineral mixtures remains comparatively under-constrained. Similar limitations apply to water datasets: AMD-impacted waters often exhibit strong gradients in acidity, turbidity, and dissolved constituents, but open data collections that combine *in situ* VNIR water spectra with co-located field measurements and laboratory analyses are still scarce.

To address these gaps, we publish a comprehensive dataset from the Kirki (Saint Philippos) mine site in NE Greece that integrates:

- Full-range laboratory mineral reflectance spectra spanning 350–15,375 nm (VNIR–SWIR–MWIR–LWIR) for 26 natural, compositionally complex AMD-related solid samples;
- For a subset of 15 of these samples, field spectra collected in the 350–2,500 nm range under natural sunlight.
- *In situ* water VNIR spectra (337–823 nm) collected from 9 streams and lakes, covering a range of acidification and turbidity conditions.
- co-located water field measurements (e.g., temperature, pH, EC, turbidity); and
- an extensive suite of laboratory analyses for both solid and water samples, including mineralogical, major- and trace-element data.

All data products, metadata, and processing notes are made openly available via the referenced Zenodo repository^15^. The dataset is designed to support a wide range of applications, including spectral algorithm development, cross-sensor comparison and calibration, environmental assessment in AMD-impacted settings, and planetary spectroscopy studies that require realistic analog materials.

## Methods

### Site description

**Fig. 1:**
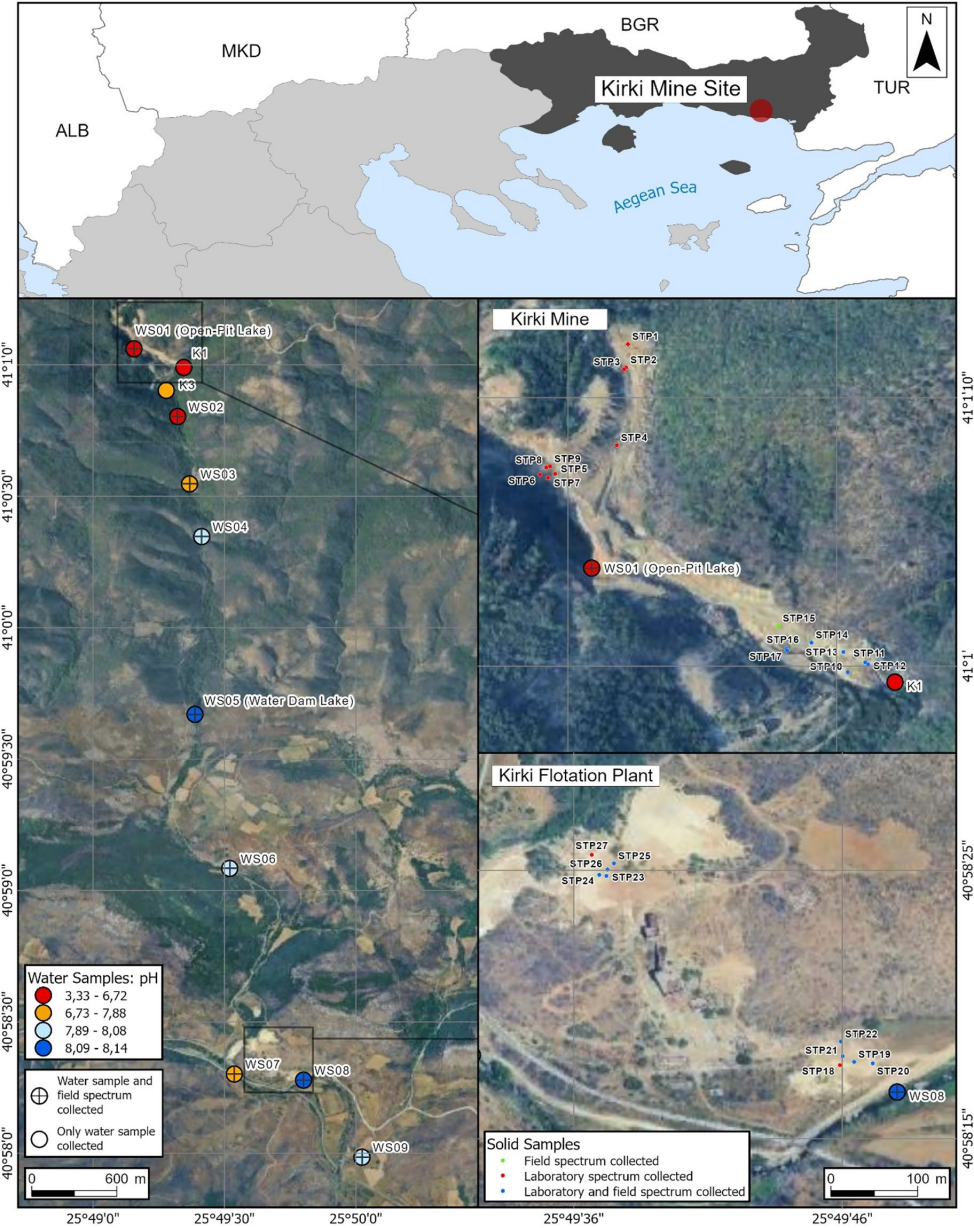
Locations of the water and solid samples. Surface water spectra were collected simultaneously with water *in-situ* measurements and samples at nine locations, where the watercourse was sufficiently wide for drone overflights. Solid samples were collected from 26 sites, for which laboratory spectra were measured and additional laboratory analyses were conducted (STP1–14, STP16–27). Unstable weather conditions restricted the acquisition of field spectra under natural sunlight to 15 of these 26 locations. In addition, one extra field spectrum (STP15) was collected specifically for image calibration and is retained in the dataset despite the absence of corresponding laboratory analyses.

The Kirki (Saint Philippos) mining site lies in NE Greece, NNW of Alexandroupolis and north of Kirki village (Fig. 1). Mining began by 1880 and continued intermittently through the 1970s–1990s, including installation of a flotation/beneficiation plant^16^. Operations created an open pit that now hosts an acidic pit lake and generated large volumes of abandoned sulfidic flotation tailings; the site was abandoned in the late 1990s, with significant environmental impacts^16–20^.

The epithermal St Philippos mineralisation is structurally controlled by NNW-trending, high-angle normal faults and occurs as breccias, crustiform massive sulfide zones, and disseminations. It is hosted within Eocene clastic sedimentary rocks (sandstones), and is spatially associated with upper Oligocene, quartz-feldspar porphyries^21^. The deposit is generally classified as a hydrothermal sulfide deposit with significant polymetallic (Fe–Cu–Pb–Zn–As–Au) mineralization overprinted by intense supergene oxidation that produces extensive acid mine drainage (AMD). The mineralisation exhibits an uncommon polymetallic character (e.g., Pb–Zn–Cu–Ag–Bi–Sn–In–U), which is well-reflected in its complex mineralogy, consisting of sulfides and several Pb–As–Cu–Ag–Bi–Sn sulfosalts^16,21–25^, and suggests that ore-deposition took place under Intermediate Sulfidation (IS) to High Sulfidation (HS) epithermal conditions^21^. Gangue minerals comprise quartz, kaolinite/dickite, sericite, pyrophyllite, Aluminium Phosphate Sulfate (APS) minerals, and barite^21^. No remediation followed closure, and the site is a persistent source of acid mine drainage (AMD) from runoff and extensive waste deposits^18,26^. AMD at Kirki shows strong acidity (pH ~1–4) and high salinity with elevated sulfate^18,26^. Metal pollution remains a long-term concern, though the Hellenic Survey of Geology and Mineral Exploration has initiated restoration efforts.

### Mineral sampling framework

During the May 2024 field campaign, various samples of solid formations were collected, with particular focus on AMD-affected areas. To optimize the number of samples in regards to geochemical patterns, *in-situ* field measurements were carried out on the same sampling sites using a portable XRF Niton XL5 Plus Handheld Analyzer, ThermoFischer. The instrument uses a 5 W X-ray tube operated in 50 kV, Ag anode and 1μm graphene window and the detection time was set to 120 s operated in soil calibration mode. Following the field portable XRF (FPXRF) measurements, surficial samples were collected from 26 selected sampling sites on the exact sites of the *in-situ* XRF measurements. Each sample was collected manually using a clean plastic shovel, comprising approximately 1 kg of loose material from a depth of 0–2 cm, and stored in a polyethylene bag. The samples were then analyzed in the laboratory, with details of the analytical methods provided in the section “Mineral samples – laboratory geochemistry.”

At the sampling sites, spectroradiometric measurements were taken under natural illumination conditions, if weather conditions allowed, using a SR-2500 portable spectroradiometer. These measurements followed the protocols detailed in the “Field-based VNIR–SWIR mineral spectral libraries” section.

Further spectral data were acquired in the laboratory from homogenized, sieved (<74 μm) samples (n = 26) under controlled conditions. These included:

- Laboratory VNIR–SWIR spectral libraries (350–2,500 nm) using the spectroradiometer’s bench top reflectance probe, detailed in the “Laboratory-based VNIR-SWIR mineral spectral libraries” section.
- Laboratory MWIR–LWIR spectral libraries (2,500–15,375 nm), detailed in the “Laboratory-based MWIR–LWIR mineral spectral libraries” section.

### Field-based VNIR–SWIR mineral spectral libraries

During the field campaign, unstable weather conditions restricted the acquisition of field spectra under natural sunlight to 15 of the 26 sampling locations (STP10–17, STP19–26, Fig. 1). One of these spectra (STP 15) was collected specifically for later image calibration. It is included in the dataset despite the absence of corresponding laboratory analyses.

Field VNIR–SWIR reflectance spectra were acquired using a Spectral Evolution SR-2500 spectroradiometer (Table 1). The sensor was consistently oriented relative to the Sun to ensure uniform illumination. Calibration to relative reflectance was performed using a 12.7 × 12.7 cm white reference panel with 100% diffuse reflectance.

**Table 1:** The specifications of the SR-2500 Spectral Evolution spectroradiometer (a three-array system).

Spectral range	350–2,500 nm
Spectral resolution	3.5 nm (350–1,000 nm)
	22 nm @ 1,500 nm
	22 nm @ 2,100 nm
Channels (resampled)	2151
Noise Equivalence Radiance defined for the three detectors	0.8 × 10^−9^ W/cm^2^/nm/sr @400 nm
	1.5 × 10^-9 ^W/cm^2^/nm/sr @1,500 nm
	1.8 × 10^-9 ^W/cm^2^/nm/sr @2,100 nm
Measurement mode	Benchtop Reflectance Probe (laboratory spectra measurement) Non-Triggering Pistol Grip (field spectra measurement)

Field spectra were collected following standard procedures: measurements were taken with a handheld, non-triggering pistol-grip spectroradiometer in nadir view, at approximately 1 m above the target, under homogeneous illumination conditions. The 25° field of view (FOV) of the fiber-optic cable corresponded to a circular footprint with a diameter of approximately 44 cm^27^.

### Mineral samples - laboratory geochemistry

**Fig. 2:**
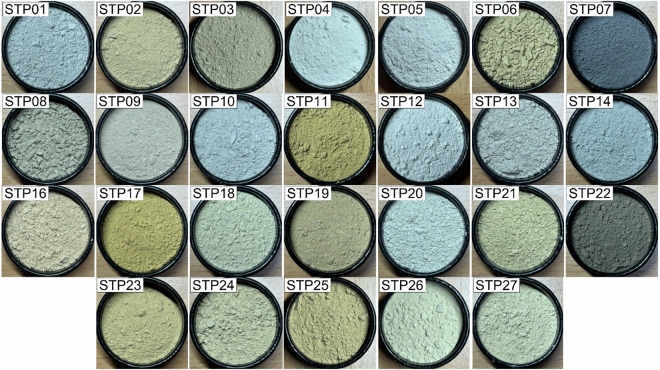
26 collected solid samples (STP1–14, STP16–27) consequently analyzed in the lab: each sample was quartered to obtain a representative subsample and ground to <74 μm (200 mesh) to prepare for detailed mineralogical and geochemical analyses conducted at the HSGME laboratories. Consequently, the laboratory-based mineral VNIR–SWIR and MWIR–LWIR spectral libraries were collected.

At the laboratories of the Hellenic Survey of Geology and Mineral Exploration (HSGME), the 26 collected samples (STP1–14, STP16–27, Fig. 2) were air-dried and manually cleared of organic residues. Each sample was quartered to obtain a representative subsample and ground to <74 μm (200 mesh) to prepare for detailed mineralogical and geochemical analyses conducted at the HSGME laboratories. The pH was measured according to the standard ISO 10390:2005 using a mixture of each material in deionized water with CaCl_2_.

The mineralogical analysis was carried out by using an XRD of PANALYTICAL X’Pert MRD PRO equipped with a CuKα radiation at 40 kV and 40 mA, step size the Savitzky-Golay filter was applied using the window size of 99 and 2nd-order polynomial configuration. 0.02°, counting time 1 sec. The obtained X-ray patterns were evaluated using the EVA 13.0 DIFFRACplus software package.

The concentrations of major elements in the form of oxides, including SiO_2_, Al_2_O_3_, Fe_2_O_3_, MgO, CaO, Na_2_O, K_2_O, TiO_2_, P_2_O_5_, and MnO, were analyzed using XRF spectroscopy with a Wavelength Dispersive X-ray Spectrometer (WDXRF) S4 PIONEER manufactured by Bruker AXS. Trace elements were analyzed using Inductively Coupled Plasma Mass Spectrometry (ICP-MS) method with a PerkinElmer SCIEX ELAN 6100 instrument.

### Laboratory-based mineral VNIR–SWIR spectral libraries

**Fig. 3:**
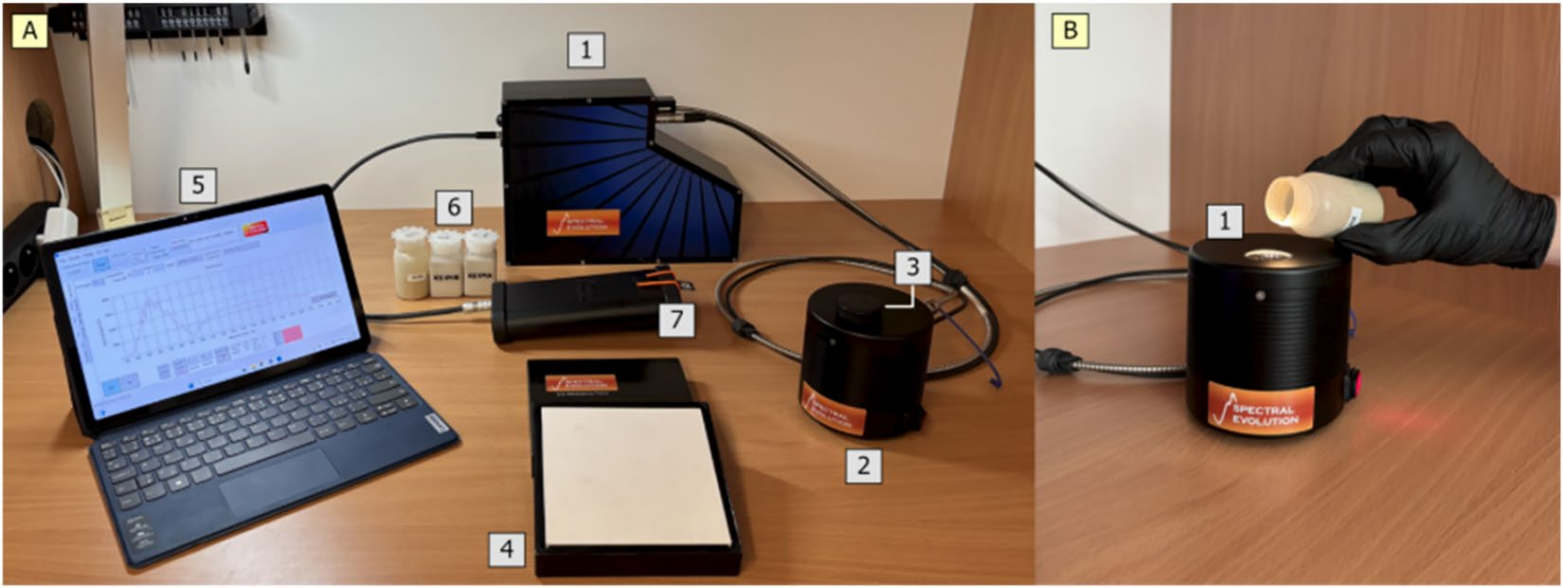
Laboratory setup for spectral measurement: (A1) Spectral Evolution SR-2500 spectroradiometer; (A2) Reflectance Benchtop; (A3) Petri dish used to cover samples and minimize external light interference; (A4) Spectralon panel for white reference calibration; (A5) DARWin LT software (version 3.0.8635) for instrument control and data acquisition; (A6) Measured samples; (A7) High-capacity battery for SR series spectroradiometers (148Whr@7.4 V); (B1) Light source: an integrated MR6 Parabolic Xenon Bulb (4.25 v, 5 W) Benchtop Reflectance Probe Flyer-BRP2-2023^55^.

The laboratory VNIR-SWIR reflectance spectra of the from the 26 powderized samples described in section “Mineral samples - laboratory geochemistry” (Fig. 2) were acquired with the Spectral Evolution SR-2500 spectroradiometer (Table 1). Laboratory reflectance measurements were acquired at the Czech Geological Survey using a Benchtop reflectance probe (laboratory setup in Fig. 3). During measurements, soil samples on the benchtop reflectance probe were covered with a black Petri dish to minimize external light interference. For each sample, four replicate measurements were acquired, each representing the average of 10 spectral readings. These four replicates were then averaged to obtain a representative spectrum per sample. The benchtop was carefully cleaned between measurements to prevent cross-contamination.

### Laboratory-based mineral MWIR–LWIR spectral libraries

**Fig. 4:**
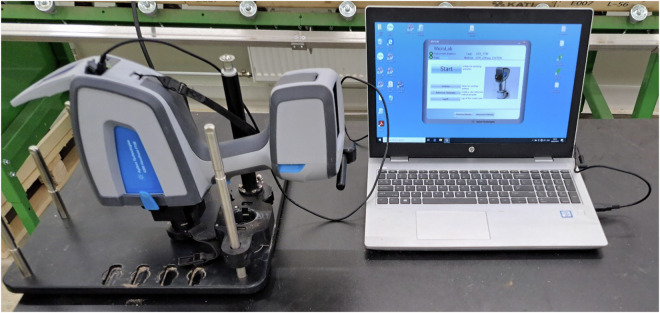
The MWIR–LWIR data acquisition setup.

The mid to long-wave infrared (MWIR–LWIR) spectral library consists of 26 spectra that were collected from the powderized samples described in section “Mineral samples - laboratory geochemistry”. These measurements were conducted at the Geological Survey of Finland using an Agilent 4300 Handheld FTIR spectrometer (Fig. 4).

The device was powered on and allowed to warm up for 30 minutes prior to data collection to ensure thermal stability and optimal performance. To ensure consistency, each sample was manually compacted into a cylindrical sample holder (30 mm in diameter and 20 mm in height) providing a uniform surface for the measurements. The effective measurement area on the sample surface had a diameter of approximately 7 mm.

Spectral data was collected in diffuse reflectance mode, with the spectrometer mounted on an adjustable stand to maintain stable and even contact with the sample surface. One spectral measurement was acquired from each sample using the settings shown in Table 2. A diffuse gold reference was used for normalization, and reference measurements were repeated every 8 minutes. The foreoptic accessory was carefully wiped clean with non-linting wipes between each measurement to prevent cross-contamination.

**Table 2:** Specifications of Agilent 4300 Handheld FTIR spectrometer.

Spectral range	2,500.36–15,374.68 nm / 3,999.43–650.42 cm^−1^
Background scans	128
Sample scans (number of individual spectra collected and then averaged for each sample measurement)	128
Spectral resolution	7.16 nm / 4 cm^−1^
Channels	1797
Apodization	Happ-Genzel function
Phase correction	Mertz phase correction algorithm
Sampling	Reflectance
Sampling subtype	Diffuse
Frequency of reference measurements	Every 8 minutes
Sample measurement diameter	7 mm

The units of the data were converted from wavenumbers to wavelengths using the Spectral Geologist (TSG version 8.1.0.5, CSIRO, Commonwealth Scientific and Industrial Research Organisation CSIRO, Canberra, Australia) software. In practice, the “dynamic L3” - resampling function of said software was used for this end. The wavenumber-to-wavelength conversion step was taken to make the data directly comparable with the spectral libraries acquired with the SR-2500 spectroradiometer as well as with the other available spectral libraries (e.g. the USGS Spectral Library)^3^.

### Full-range mineral (VNIR–SWIR–MWIR–LWIR) spectral libraries

**Fig. 5:**
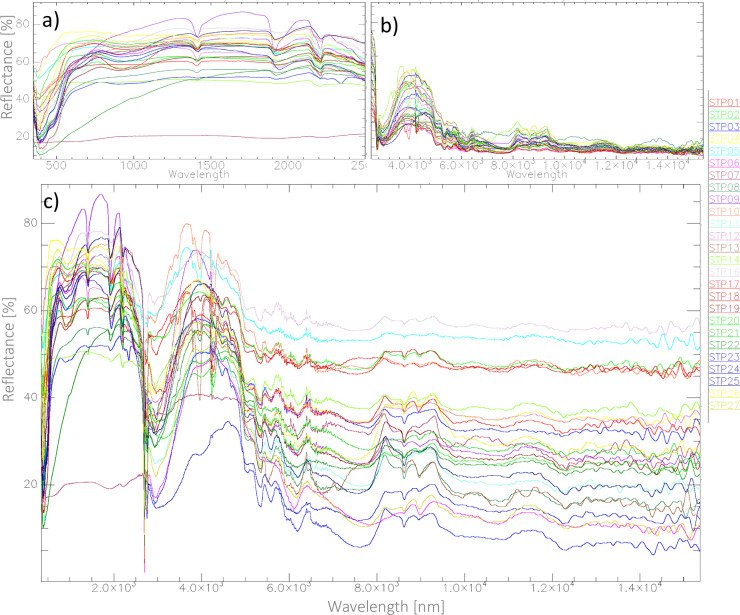
Mineral spectral libraries: (**a**) VNIR–SWIR reflectance spectra (SR-2500 spectrometer); (**b**) MWIR–LWIR reflectance spectra (Agilent 4300 Handheld FTIR spectrometer); (**c**) a full-range spectral dataset (350-15,375 nm) with corrected detector-gap between SR-2500 and Agilent 4300 Handheld FTIR spectrometer. Wavelength in nm.

Mineral spectral libraries (VNIR-SWIR and MWIR–LWIR) were merged to form a full-range (350–15,375 nm) mineral spectra with corrected detector-gap between SR-2500 and Agilent 4300 Handheld FTIR spectrometer (Fig. 5). The detector-gap correction factor between the optical libraries acquired with the SR-2500 (350–2,500 nm) and those acquired with the Agilent 4300 Handheld FTIR spectrometer (2,500–15,375 nm) was calculated as the difference between the reflectance values at the boundary wavelengths. Specifically, it is the difference between the SR-2500 reflectance at 2,500 nm (the last wavelength recorded by the SR-2500) and the Agilent 4300 reflectance at 2,500.36 nm (the first wavelength recorded by the Agilent 4300).

The correction factor C is:

$${\rm{C}}={{\rm{R}}}_{{\rm{SR}}}(2,500.00\,{\rm{nm}})-{{\rm{R}}}_{{\rm{A}}}(2,500.36\,{\rm{nm}})$$

R_SR_(λ) denote the reflectance measured by the SR-2500 at wavelength 2,500.00 nm, and R_A_(λ) denote the reflectance measured by the Agilent 4300 at wavelength 2,500.36 nm.

### Water sampling and *in-situ* data acquisition

The water sampling campaign in the Kirki mining area was conducted between May 28–30, 2024, to capture a broad range of acidification levels and better characterize AMD effects. Sampling sites were pre-selected to align with spectral measurements, ensuring complementary datasets. In total, 18 samples were collected from the Kirkalon and Eirini streams, as well as from the open-pit lake and the water-dam lake along Kirkalon (see Fig. 1).

Samples from the Kirkalon and Eirini streams were collected by hand, while sampling from the highly acidic open-pit lake (WS01) and steep-edged dam lake (WS05) was performed using a WALKERA V1100 PRO drone. For the dam lake, a DROSENS water sampler (1.5 L) was attached to the drone and lowered to the target depth with a remotely operated winch (Foxtech F10). The sampler was triggered via a mobile application using a Bluetooth connection. In the open-pit lake, the water was too acidic for the Bluetooth sensor to function, so a standard plastic measuring jug was attached to the winch.

Simultaneously with sample collection, *in-situ* water quality measurements were performed using a YSI ProDSS Multiparameter instrument. Parameters measured included temperature, pH, oxidation–reduction potential (ORP), electrical conductivity (EC), total dissolved solids (TDS), dissolved suspended solids (DSS), turbidity, and dissolved oxygen (DO). The sonde was rinsed twice with the sample water before measurements. The sample was then poured into the measurement cup, and readings were allowed to stabilize. Measurements were recorded in continuous mode every four seconds, and average values were calculated for the dataset.

At each site, 500 mL of unfiltered, untreated water was collected in polypropylene containers for major element analysis and subsequently submitted for consequent laboratory analysis. In addition, an aliquot of 50 mL from each sample was filtered *in situ* using 0.45 μm filter units. Acid mine drainage (AMD) water samples were collected in polypropylene containers pre-washed with 10% HNO₃ and subsequently acidified with ultrapure HNO₃ to prevent metal precipitation. A second, unfiltered aliquot (no acidification) was collected in 100 mL glass bottles pre-washed with dilute HCl for 24 hours and stored immediately in a portable refrigerator for total organic carbon (TOC) and dissolved organic carbon (DOC) analyses.

### Field-based VNIR water spectral libraries

**Fig. 6:**
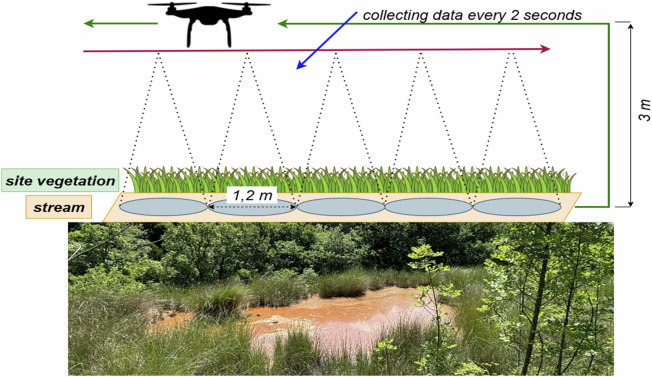
AMD site located at the WS02 point: acquisition of field-based VNIR water spectral libraries.

Surface water spectra were collected simultaneously with water sampling using an Ocean Optics STS-VIS spectrometer (Table 3) mounted on a DJI Phantom 3 drone. Measurements were taken at nine locations situated between the Kirki Mine and the Kirki Flotation Plant, where the watercourse was sufficiently wide for drone overflights (Fig. 1). At each site, spectra were acquired from a height of three meters above the water surface, providing a spatial resolution of approximately 1.2 meters for each measurement spot. The data collection followed a consistent pattern: 50 meters downstream, 50 meters upstream, and diagonally across the stream, with spectra recorded every two seconds^28^ (Fig. 6).

**Table 3:** Specification of the Ocean Optics STS-VIS spectroradiometer.

Dimensions	40 × 42 × 24 mm
Spectral range	337–823 nm
Spectral resolution	1.2 nm
Channels	1024
Field of view (FOV)	25°

To calculate relative reflectance, the spectrometer measured the intensity of a white reference panel with 100% diffuse reflectance and a dark spectrum. These reference values, together with the measured water signal, were used to derive relative reflectance based on the formula provided in the OceanView installation and operation manual (2013)^29^.

To enhance the quality of the spectral data and minimize the impact of noise, particularly caused by the low reflectance and the high spectral resolution of the spectroradiometer, the Savitzky-Golay filter was applied using the window size of 99 and 2nd-order polynomial configuration. This smoothing technique operates by fitting successive subset of adjacent data points with a low-degree polynomial using the least-squares method, effectively preserving important spectral features while reducing random fluctuations^30^.

Due to the large volume of water spectra collected continuously every 2 seconds at each site, automatic preprocessing was applied to improve data reliability. For each location, we calculated the mean spectrum and standard deviation (SD), then excluded any individual spectra that exceeded a predefined threshold of 1.5 SD. This procedure was applied iteratively three times to ensure consistent outlier removal. The remaining spectra were then averaged to produce a single representative spectrum per site. Finally, a spectral library was compiled from the resulting processed mean spectra for nine selected water sampling locations.

### Water samples - laboratory geochemistry

For the quantification of Total Organic Carbon (TOC) and Dissolved Organic Carbon (DOC) content, the standard method ISO 8245:1999 was followed using a Shimadzu TOC-VCSH analyzer. The laboratory water pH and Electrical Conductivity (EC, μS cm^−1^, 25 °C) were measured by a WTW multi-meter type 740 InoLab and a WTW conductivity meter type 730 InoLab, respectively. Chemical parameters including sulfates (SO_4_^2−^), nitrates (NO_3_^−^), nitrites (NO_2_^−^) and ammonium (NH_4_^+^) were quantified using a Hach DR6000 UV–VIS spectrophotometer. The HCO_3_^−^, CO_3_^2−^ and Cl^−^ content was determined using potentiometric titrations with Metrohm 808 Titration system combined with an 814 USB Sample Processor.

The Ca, Mg, Na, K and Fe contents were determined with inductively coupled plasma optical emission spectrometry (ICP-OES) method using a ICP-OES Perkin Elmer Optima 5300 DV, while the dissolved trace elements (SiO_2_, Ag, Al, As, Ba, Bi, Cd, Co, Cr, Cu, Fe, Li, Mn, Ni, Pb, Sb, Se, Sr, U, Zn) were determined with a PerkinElmer NexION 2000 ICP-MS spectrometer.

## Data Records

**Fig. 7:**
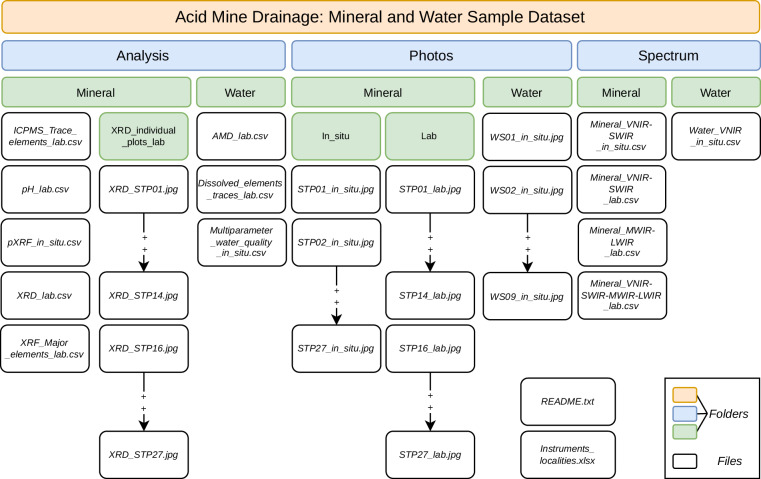
Structure of the dataset, including spectral data (minerals and water), *in situ* measurements, laboratory analyses, and illustrative photographs. A total of 26 samples were analyzed in the laboratory, numbered STP1–STP14 and STP17–STP26. An additional field spectrum (STP15) was collected specifically for image calibration and is retained in the dataset despite the absence of corresponding laboratory analyses. Consistent sample naming is maintained across all datasets to ensure mutual correspondence.

The datasets described above have been deposited on Zenodo^15^. Spectra (mineral and water) are located in the respective subfolders under the Spectrum folder as.csv files. Mineral and water laboratory analyses are provided in the Analysis folder under the respective subfolders as.csv files. Additionally, XRD lab data are provided as.jpg plots representing each sample ID. Dataset naming within all folders corresponds to the respective analytical method used and the dataset origin (laboratory or *in-situ*), as shown in the Database schematic structure (Fig. 7). Photographs of each sample (mineral or water, lab or *in-situ*), complementing the data records, are named using the respective Sample ID and are located in the Photos folder. Used instrument details for all analytical and spectral datasets are summarized in Instruments_localities.xlsx within the main folder. Information about project, funding and dataset licencing are presented in the README.txt file.

## Technical Validation

### AMD mineral patterns

To assess the internal consistency of the solid-phase dataset, we compared the laboratory geochemical analyses with established conceptual models for AMD systems. Acid mine drainage environments commonly exhibit strong gradients in pH, redox conditions, and mineral stability from source zones to downstream areas, accompanied by systematic changes in sulfur and iron contents and associated secondary minerals. Similar AMD behaviour has previously been reported at the Kirki site^10,11,31^, including the development of ferric hydroxides and oxyhydroxysulfates under acidic, oxidizing conditions and more goethite-dominated assemblages at higher pH.

**Fig. 8:**
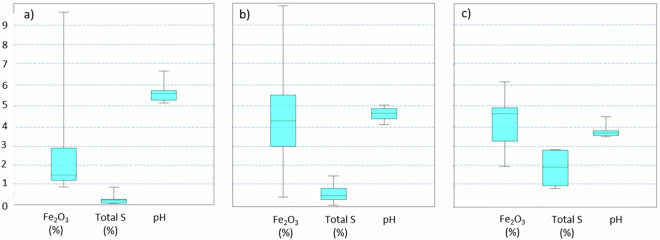
Total S and Fe₂O₃ concentration gradients at the Kirki site as a function of pH: (**a**) Total sulfur S and Fe₂O₃ contents under near-neutral to neutral conditions (pH 5–7); (**b**) Total S and Fe₂O₃ contents under acidic conditions (pH 4–5); and (**c**) Total S and Fe₂O₃ contents under very acidic conditions (predominantly pH < 4).

**Fig. 9:**
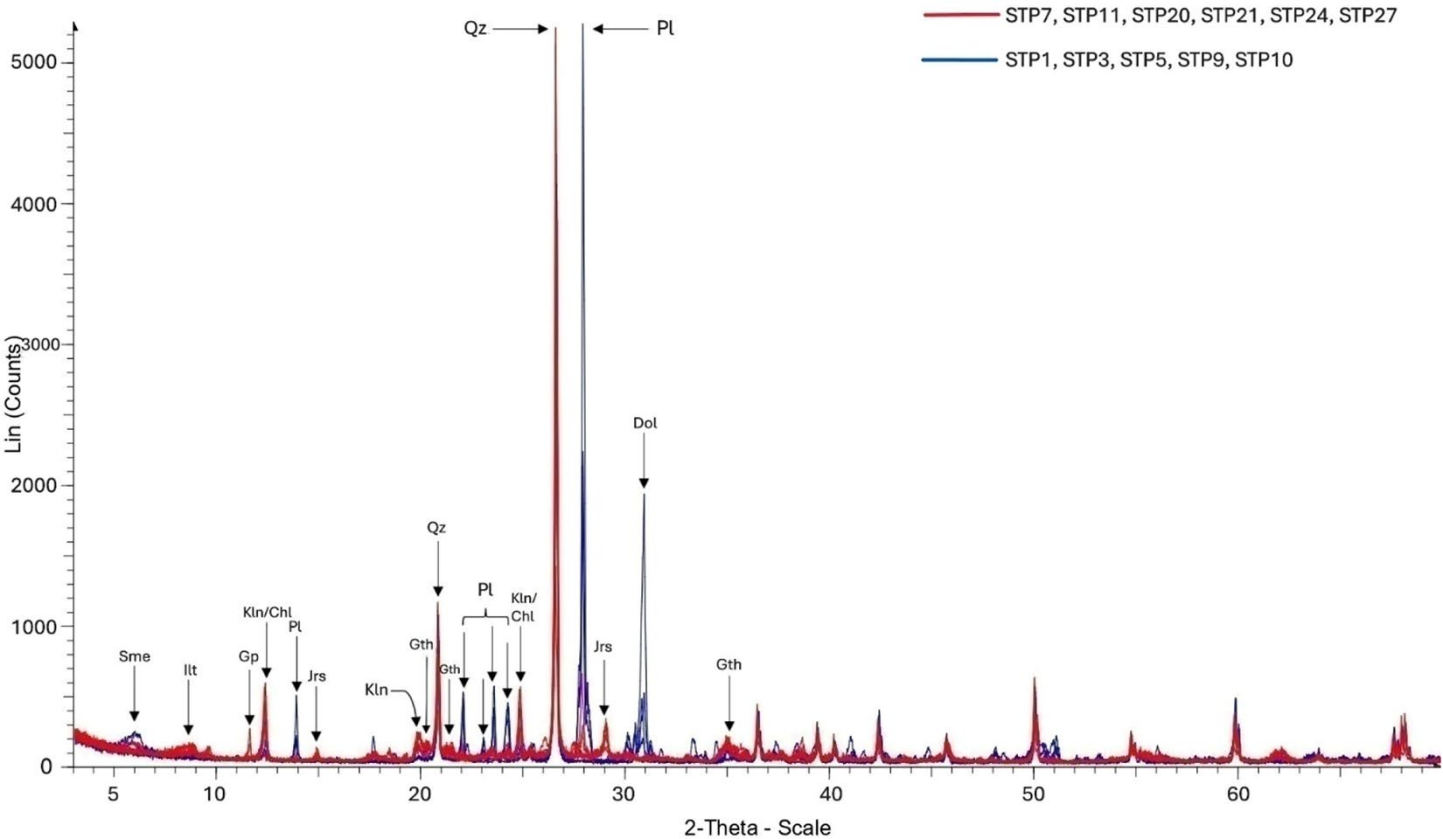
XRD patterns of selected samples from the Kirki site. Samples are color-coded: in red those with pH values < 4 and in blue those with pH values > 5. The minerals identified in the samples include: Dol - Dolomite, Gth - Goethite, Gp - Gypsum, Ilt - Illite, Kln - Kaolinite, Jrs - Jarosite, Pl - Plagioclase, Qz - Quartz, and Sme - Smectite. The abbreviations for mineral names follow Warr (2021)^56^.

In our dataset, bulk geochemical parameters (e.g., total sulfur (S), Fe_2_O_3_) were examined as a function of pH, and XRD results were checked for consistency with these trends. Samples from more acidic locations contain higher total S and Fe₂O_3_ and show XRD patterns dominated by sulfates and ferric oxyhydroxysulfates, whereas samples from less acidic sites show reduced sulfur contents and increased proportions of iron oxyhydroxides and silicates (Figs. 8, 9). These patterns are consistent with previously described AMD weathering sequences.

### Spectral-geochemical consistency

For the full-range mineral spectra (350–15,375 nm), we used partial least squares regression (PLSR)^32–34^, a standard chemometric method, as a quantitative consistency check between spectra and bulk composition. We focused on Fe₂O₃, total S and SiO₂ as these are key parameters for the Kirki AMD system (Figs. 8 and 9).

**Fig. 10:**
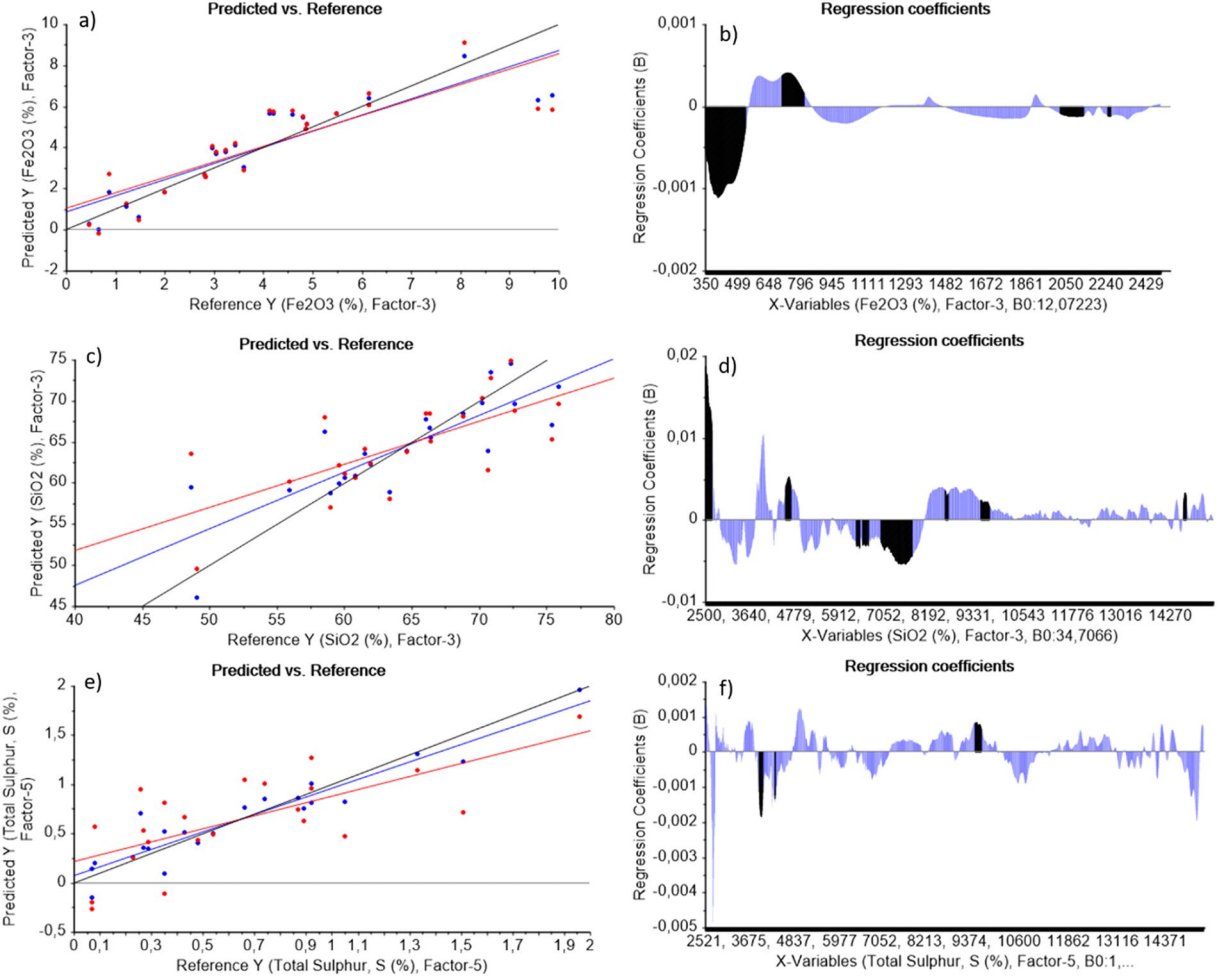
PLSR models (Cal: blue, Val: red) and coefficients constructed for the selected geochemical parameters: (**a,b**) Fe_2_O_3_,(**c,d**) SiO_2_,(**e,f**) Total S.

**Fig. 11:**
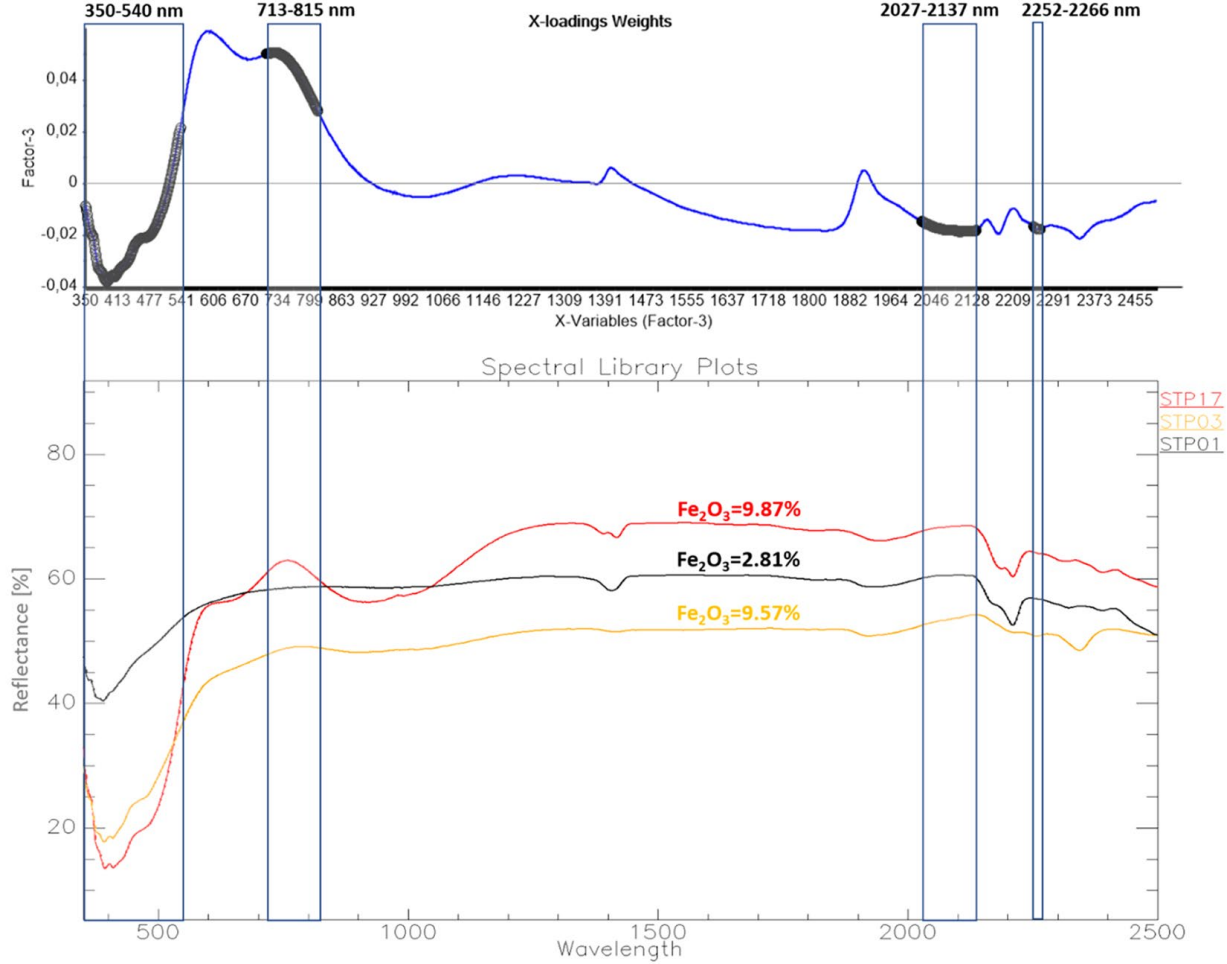
PLSR variable-importance profile for Fe_2_O_3_, showing spectral regions that contribute to the example regression model and are consistent with known diagnostic features of Fe-bearing minerals. Wavelength in nm.

**Fig. 12:**
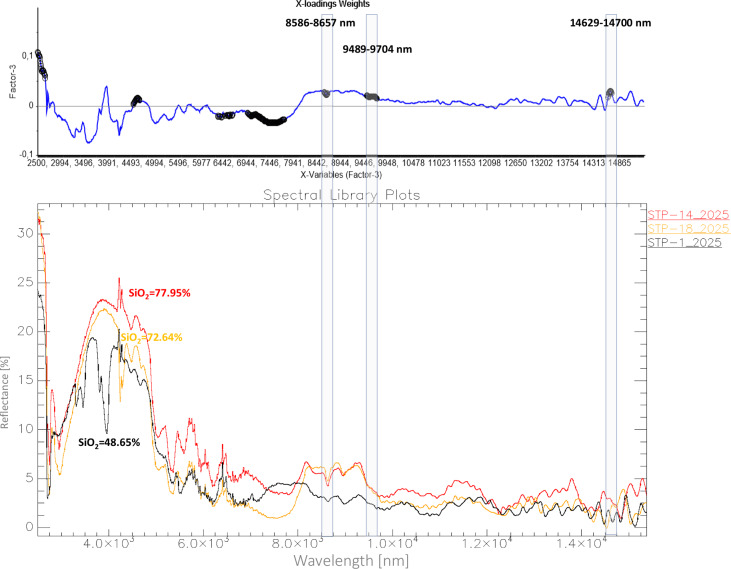
PLSR variable-importance profile for SiO_2_, showing LWIR spectral regions that contribute to the example regression model and are consistent with known silicate diagnostic features. Wavelength in nm.

**Fig. 13:**
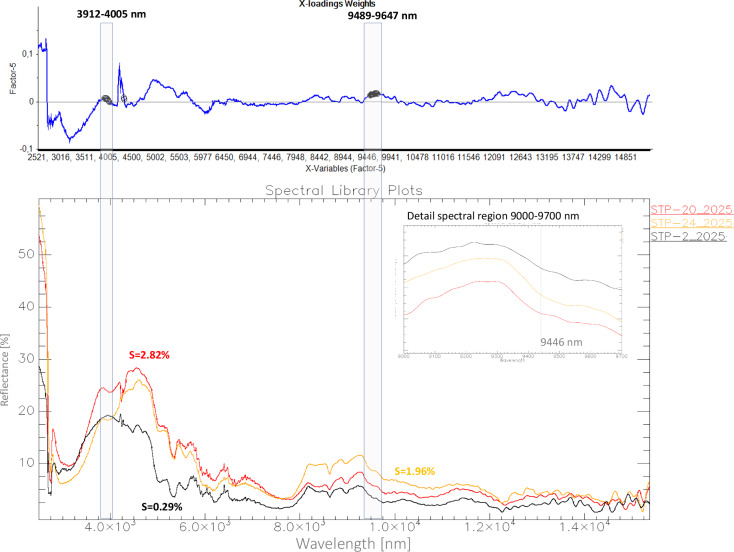
PLSR variable-importance profile for Total S, showing spectral regions that contribute to the example regression model and are consistent with expected sulfate-related features. Wavelength in nm.

PLSR models were built for three input spectral configurations: (i) full-range spectra, (ii) VNIR–SWIR spectra, and (iii) MWIR–LWIR spectra. Model robustness was assessed using leave-one-out cross-validation, reporting coefficients of determination (R^2^) and root-mean-square errors (RMSE) for calibration (Cal) and validation (Val) (Table 4). The regression coefficients (Fig. 10) and variable-importance measures (Figs. 11–13) were then examined to identify spectral regions that contribute most strongly to each model.

**Table 4:** Best-performing PLSR models for selected geochemical parameters. For each parameter, the spectral ranges identified as most important are also reported.

Geochemical parameter	R^2^ Cal/Val	R Cal/Val	Number of factors	RMSE Cal/Val	Spectral range (nm)
Fe_2_O_3_	0.73/0.71	0.86/0.82	3	1.281/1.400	350–2500
Total S	0.89/0.48	0.94/0.70	5	0.163/0.369	2,500–15,375
SiO_2_	0.69/0.52	0.83/0.69	3	4.050/5.270	2,500–15,375

For Fe₂O₃, the most influential wavelengths occur in VNIR–SWIR regions commonly associated with ferric iron absorption features and Fe-oxide/oxyhydroxysulfate behaviour (Fig. 11). For SiO₂, important wavelengths are located in MWIR–LWIR Reststrahlen and bending regions diagnostic of silicate frameworks (Fig. 12). For total S, PLSR highlights spectral regions where sulfate-bearing phases are known to exhibit combination and fundamental bands (Fig. 13). These patterns are consistent with the independent XRD phase identifications and bulk oxide data, indicating that the spectral measurements and geochemical analyses are mutually coherent and suitable for quantitative analyses by data users. In addition, we provide a detailed overview of important spectral regions (identified by the PLSR) and their diagnostic interpretations, together with relevant references, for Fe₂O₃ (Table 5), SiO₂ (Table 6), and total S (Table 7).

**Table 5:** Fe_2_O_3_: Important spectral region depicted by the PLSR and their possible interpretations.

Important spectral region depicted by the PLSR models (Fig. 11)	Diagnostic feature possible interpretation^3,4,47–49^	PLSR statistical relationship
350–540 nm	~0.43–0.46 µm: Fe³^+^ ligand-to-metal charge-transfer edge (very strong UV–blue absorption) driving red/yellow colors.	Low negative coefficient values/negative correlation The deeper the absorption is the higher is the Fe_2_O_3_ content
713–815 nm	Shoulder between the 0.6–0.7 µm and shallow feature ~0.86–0.92 µm: (Fe³^+^ electronic transition, ferric oxides/hydroxides).	High positive coefficient values/positive correlation The higher the shoulder is the higher is the Fe_2_O_3_ content.
2,027–2,137 nm	Right shoulder of the absorption feature ~1.85–1.93 µm; (H₂O) and sulfate-related	The steeper the slope between 2,027–2,137 nm is, the lower is the Fe_2_O_3_ content.
2,252–2,266 nm	Sulfate-related overtones/combination tones near ~2.25–2.45 µm (mineral-specific)	Low negative coefficient values/negative correlation. The deeper the absorption is the higher is the Fe_2_O_3_ content.

**Table 6:** SiO_2_: Important spectral region depicted by the PLSR and their possible interpretations.

Important spectral region depicted by the PLSR models (Fig. 12)	Diagnostic feature possible interpretations^3,7,50^
8,586–8,657 nm / 8.59–8.66 µm	Near the shortwave side of the main Si–O asymmetric stretch Reststrahlen region. Strong for SiO₂ polymorphs (quartz ~8.4–8.6 µm) but also present for framework silicates. Indicates silica-rich frameworks: quartz and feldspars, with quartz typically at slightly shorter wavelengths than many feldspars.
9,489–9,704 nm / 9.49–9.70 µm	Within the main Si–O stretch Reststrahlen region for chain and sheet silicates characteristic of: • Pyroxenes (orthopyroxene/clinopyroxene often peak ~9.3–10.2 µm) • Amphiboles (broad, multi-lobed ~9.2–10.5 µm) • Many phyllosilicates (micas/clays) have peaks in ~9.0–10.0 µm too Less typical for pure quartz, has a strong asymmetric Si-O stretching band near 8.4–8.6 µm.
14,290–14,700 nm / 14.29–14.70 µm	• Long-wavelength bending region; for many silicates, the Si–O–Si bending fundamentals or combination bands extend toward or beyond 14 µm. Strongly expressed in chain silicates: • Pyroxenes commonly have features approaching ~14 µm. • Amphiboles can also carry energy into this region.

**Table 7:** Total S: Important spectral region depicted by the PLSR and their possible interpretations.

Important spectral region depicted by the PLSR models (Fig. 13)	Diagnostic feature explanation^34,51–54^
3,912–4,005 nm / 3.912–4.005 µm	For S-bearing phases: • Some sulfates (e.g., gypsum, anhydrite, alunite, jarosite) exhibit weak combination/overtone bands in the 3.8–4.2 µm neighbourhood related to SO_4_^2−^ vibrations.
9,489–9,647 nm / 9.489–9.647 µm	Consistent with sulfate signatures. It can also be influenced by co-existing silicates (pyroxenes, amphiboles, feldspars) whose Reststrahlen lie nearby, but the overlap with known SO_4_ ν3 positions.

For the water dataset, we carried out basic plausibility checks between *in situ* field measurements, laboratory analyses, and VNIR drone-based spectra (337–823 nm). Field parameters (e.g., pH, electrical conductivity, turbidity) show ranges and combinations typical for AMD-impacted streams and lakes^35^. After the preprocessing described in the “Field-based VNIR water spectral libraries” chapter, the water spectra from individual locations show reproducible shapes and level offsets that are consistent with the *in-situ* and laboratory measurements (e.g., clearer versus more turbid waters). This agreement between spectral characteristics, *in situ* measurements, and laboratory data supports the reliability of the water spectral library for subsequent independent analyses.

**Fig. 14:**
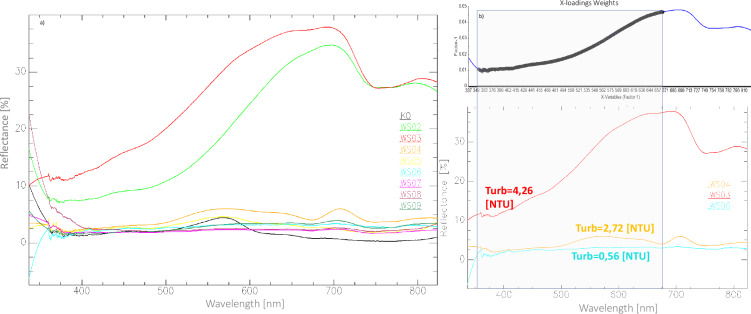
(**a**) VNIR water spectral libraries (Ocean Optics STS-VIS spectrometer); (**b**) PLSR variable-importance profile for Cd defined by PLSR, illustrating that the water spectra contain information consistent with the co-measured turbidity (Turb, in nephelometric turbidity units, NTU). Intended as a demonstration of dataset usability rather than a comprehensive analysis.

In addition to the qualitative checks described above, we performed an illustrative PLSR analysis linking VNIR water spectra (337–823 nm) to dissolved Cadmium (Cd) concentrations. This choice was motivated by the pronounced Cd gradients in the Kirki water dataset and by the fact that Cd is a highly mobile and toxic trace metal typically associated with polymetallic sulfide mineralization and AMD. Despite the limited number of sites (n = 9) cross-validated models achieved reasonable agreement between predicted and measured concentrations (R^2^Cal = 0.75, R^2^Val = 0.59). Variable-importance analysis highlights the 350–670 nm region as particularly influential (Fig. 14), which is consistent with the known sensitivity of this part of the spectrum to water color and turbidity^36–40^. This example is intended solely as a demonstration that the water spectra can support quantitative relationships with co-measured chemical parameters; it does not represent a full interpretation of Cd transport or geochemical processes at the site. Users are encouraged to explore alternative models and additional parameters using the open dataset.

### Method limitations

The processing workflow inevitably involves trade-offs that may influence data quality, and we therefore briefly outline their implications. In particular, the grinding and homogenization steps adopted to ensure representative, optically isotropic powders can modify the physical state of some phases, especially delicate or poorly crystalline clay minerals^41^. Prolonged or aggressive grinding may partially disrupt clay textures, alter particle-size distributions, or affect interlayer hydration, which in turn can slightly shift or broaden diagnostic absorption features^42^. We mitigated these effects by using standardized, relatively gentle grinding conditions and by minimizing processing time, but users should remain aware that the spectra represent well-mixed, fine-powder end-members rather than intact field textures. For applications that are highly sensitive to micro-texture or to the precise expression of clay features (e.g., detailed crystallinity assessments or comparison with minimally processed outcrop spectra), these potential impacts should be considered when interpreting the data.

In case of using the full-range spectral library (350–15,375 nm) user should be aware that loose VNIR–SWIR samples and compacted MWIR–LWIR pellets are expected to differ in their detailed spectral expression, particularly in terms of absolute reflectance, scattering behavior, and the contrast of certain features. In our workflow, these different configurations were chosen to optimize signal quality in each spectral range (minimizing specular reflection and maximizing signal-to-noise ratio (SNR) in the VNIR–SWIR, and ensuring stable emissivity/reflectance geometry in the MWIR–LWIR). As a consequence, the full-range spectra should be viewed primarily as wavelength-continuous, compositionally consistent references, rather than as strict photometric analogues of a single physical surface state. Users who are sensitive to surface-texture effects (e.g., bidirectional reflectance distribution function (BRDF)) studies or detailed radiative-transfer modelling) should therefore be aware that differences in packing and porosity between the loose and compacted configurations may introduce modest changes in band depths and continuum shape, even though the positions and identities of diagnostic features are preserved.

## Usage Notes

Beyond the applications demonstrated here, the Kirki AMD datasets can support a wide range of future research directions. The full-range mineral spectra and co-registered geochemistry enable development and benchmarking of new spectral indices, machine-learning models, and unmixing approaches for complex mixtures, as well as forward and inverse radiative-transfer studies that explicitly account for grain size, coatings, hydration, and texture. The VNIR water spectra, combined with detailed hydrochemistry, provide a testbed for refining optical proxies of acidity^43,44^, turbidity, and trace-metal behaviour and for exploring multi-sensor data fusion (e.g., drone, airborne, and satellite observations) in mine-impacted catchments^45,46^. In planetary science, the AMD mineral assemblages and LWIR signatures offer realistic analogs for sulfate- and Fe-oxide–bearing terrains, supporting instrument design, band-selection studies, and cross-calibration of VNIR–SWIR–TIR missions. More broadly, the dataset can underpin comparative studies of mine-waste remediation, automated anomaly detection in hyperspectral surveys, and teaching or training activities that require openly available, fully documented spectral–geochemical reference material.

No specific software is required to open the datasets as they are available as csv or jpg files.

## Data Availability

The datasets described in the manuscript have been deposited on Zenodo: [https://zenodo.org/records/17409854].

## Code availability

Python code for spectral data (collected using STS-VIS Spectrometer) preprocessing. The distribution version of the code can be obtained at the following website: https://github.com/martinkyhos/Acid-Mine-Drainage-AMD-Mineral-and-Water-Sample-Datasets.git.

The authors declare that no other custom code was generated or used in this work.
